# Unique molecular signatures of microRNAs in ocular fluids and plasma in diabetic retinopathy

**DOI:** 10.1371/journal.pone.0235541

**Published:** 2020-07-21

**Authors:** Zeljka Smit-McBride, Anthony T. Nguyen, Alfred K. Yu, Sara P. Modjtahedi, Allan A. Hunter, Saadia Rashid, Elad Moisseiev, Lawrence S. Morse

**Affiliations:** Department of Ophthalmology & Vision Science, Vitreoretinal Research Laboratory, School of Medicine, University of California Davis, Davis, California, United States of America; University of Florida, UNITED STATES

## Abstract

The main objective of this pilot study was to identify circulatory microRNAs in aqueous or plasma that were reflecting changes in vitreous of diabetic retinopathy patients. Aqueous, vitreous and plasma samples were collected from a total of 27 patients undergoing vitreoretinal surgery: 11 controls (macular pucker or macular hole patients) and 16 with diabetes mellitus(DM): DM-Type I with proliferative diabetic retinopathy(PDR) (DMI-PDR), DM Type II with PDR(DMII-PDR) and DM Type II with nonproliferative DR(DMII-NPDR). MicroRNAs were isolated using Qiagen microRNeasy kit, quantified on BioAnalyzer, and profiled on Affymetrix GeneChip miRNA 3.0 microarrays. Data were analyzed using Expression Console, Transcriptome Analysis Console, and Ingenuity Pathway Analysis. The comparison analysis of circulatory microRNAs showed that out of a total of 847 human microRNA probes on the microarrays, common microRNAs present both in aqueous and vitreous were identified, and a large number of unique microRNA, dependent on the DM type and severity of retinopathy. Most of the dysregulated microRNAs in aqueous and vitreous of DM patients were upregulated, while in plasma, they were downregulated. Dysregulation of miRNAs in aqueous did not appear to be a good representative of the miRNA abundance in vitreous, or plasma, although a few potential candidates for common biomarkers stood out: let-7b, miR-320b, miR-762 and miR-4488. Additionally, each of the DR subtypes showed miRNAs that were uniquely dysregulated in each fluid (i.e. aqueous: for DMII-NPDR was miR-455-3p; for DMII-PDR was miR-296, and for DMI-PDR it was miR-3202). Pathway analysis identified TGF-beta and VEGF pathways affected. The comparative profiling of circulatory miRNAs showed that a small number of them displayed differential presence in diabetic retinopathy vs. controls. A pattern is emerging of unique molecular microRNA signatures in bodily fluids of DR subtypes, offering promise for the use of ocular fluids and plasma for diagnostic and therapeutic purposes.

## Introduction

Diabetic retinopathy (DR), diabetic macular edema and associated conditions, are the leading and growing causes of vision impairment and blindness in the United States and throughout the world. Current management involves laser therapy, intravitreal injections using anti-VEGF, anti-inflammatory, steroid therapeutics and possible intraocular surgery. These interventions alleviate issues only temporarily and often require repeated invasive treatments. Furthermore, some patients do not respond well to current therapies. Thus, the need for new therapeutic targets and approaches is clear and compelling.

Recently, a novel type of RNA, microRNA (miRNAs), has been implicated in human diseases [1, 2]. MiRNAs are a class of small non-coding RNAs that regulate gene expression at the posttranscriptional level by either degrading or blocking translation of messenger RNA targets [3]. Besides their presence in tissues, miRNAs circulate in the bloodstream in a highly stable, extracellular form and are being investigated as blood-based biomarkers for cancer and many other diseases [4]. Identification and characterization of DR on the level of miRNA and their target molecular pathways could lead to novel diagnostic tools as well as therapies to prevent and reverse vision loss for these patients.

Circulatory miRNAs have been shown to be differentially expressed in diabetics in serum and plasma studies [5], urine [6], retina and retinal endothelial cells (RECs) of streptozocin (STZ)-induced diabetic rats [7]. Not enough is known about miRNA profiles of ocular fluids, and further research needs to be done. Therefore, we conducted a small scale preliminary study in order to evaluate feasibility of the key steps in a future, full-scale project. In this pilot study, we attempted to address the critical immediate problem—the identification of biological markers of retinal disease in diabetic retinopathy. Our goal was to identify biomarkers that are present in aqueous, vitreous and plasma, with the ultimate goal of identifying early biomarkers for progression from non-proliferative to proliferative DR. Ideally, identifying plasma biomarkers that correlate with ocular biomarkers and different stages of DR would allow the most accessible fluid (plasma) for sampling to follow changes in miRNA dynamics in the eye. A secondary goal would be to identify aqueous biomarkers that correlate with vitreous biomarkers that would allow sampling of the more accessible fluid (aqueous humor) that may correlate better with retinal pathology.

## Materials and methods

### Human samples

The samples were from the Clinical trial “Study of Ocular Fluid, Serum for Biomarkers of Eye Disease in Patients,” UC Davis Institutional Review Board (IRB) approved (IRB #216607), a single-site, investigator-initiated clinical study in the Department of Ophthalmology, UCD. The research was conducted in accordance with the 1964 Helsinki Declaration. Sample collections took place at surgery point-of-care and medical records were accessed 06/2011-04/2014. These were patients that had sought treatment at the clinic at UCD Ophthalmology for several years (01/2000-current). The dates of surgery were during the period of 3 years, 06/2011-04/2014. Very limited data from their medical record was used, and that use was covered by informed consent. These samples have a representation of both genders and various race groups. Age ranges were from 30-80yr old. To assure the anonymity and protection of human subjects, the samples were identified by acquisition number and a record of chronological age, gender, and a description of the case. **Inclusion criteria:** Patients undergoing vitrectomy surgery for retinal disorders (macular puckers, macular holes, tractional retinal detachments). **Exclusion criteria:** Prior vitreous or retinal detachment surgery, prior history of uveitis, endophthalmitis, prior intraocular injections with steroids or anti-VEGF agents, prior cataract surgery less than 6 months before inclusion, no open posterior capsules, no recent vitreous hemorrhage within 6 months, penetrating trauma, ruptured globe repair, intraocular tumor, systemic disease including cancer, connective tissue disease, current use of systemic steroids or immune-modulating agents.

### Experimental design

There were 4 groups of patients: **Group 1**: Controls were patients without diabetes who were undergoing macular hole surgery or macular pucker surgery (CON), n = 11 patients (30 samples); **Group 2**: Diabetes mellitus, Type I with proliferative diabetic retinopathy (DMI-PDR), n = 5 patients (13 samples); **Group 3**: Diabetes mellitus, Type II with proliferative diabetic retinopathy (DMII-PDR); n = 7 patients (18 samples); and **Group 4**: Diabetes mellitus, Type II with non-proliferative diabetic retinopathy (DMII-NPDR), n = 4 patients (12 samples). We collected samples from 27 patients, and for each patient, with rare exceptions, we had 3 samples from different bodily fluids (aqueous, vitreous and plasma), with a total of 73 samples (**S1 Table**).

### Sample collection, isolation of miRNAs, and quality control

Aqueous and vitreous humor, as well as plasma samples, have been collected from DM and control patients during the standard-of-the-care eye surgery. Samples of 100-200ul were collected, aliquoted in 100ul aliquots, frozen on dry ice immediately and stored at -80°C. RNA was isolated using Exiqons’ modification of Qiagen’s microRNeasy kit. Quantification and quality check of isolated miRNA was performed on BioAnalyzer (Agilent) with Small RNA microfluidics Chips.

### MicroRNA Affymetrix microarray probe labeling

Probe labeling was done from 10 ng of aqueous, 20ng of vitreous and 20ng of plasma miRNA samples from a total of 24 aqueous, 24 vitreous and 25 plasma samples using FlashTag (Affymetrix) procedure. Labeled probes were hybridized to Affymetrix miRNA Array 3.0 in UC Davis Genome Center Microarray Core Facility using the standard procedure (Affymetrix, Santa Clara, CA). The total data set included 73 Affymetrix miRNA 3.0 microarrays. Upon scanning of the hybridized and washed Chips, data was obtained in a set of Affymetrix data files (.cel,.arr,.jpg). This protocol has been deposited at protocols.io, and it has been assigned its own identifier (DOI): dx.doi.org/10.17504/protocols.io.bc7pizmn.

### Microarray data analysis

The data sets in.cel files were analyzed with the Expression Console and Transcriptome Analysis Console (Affymetrix, Santa Clara). The analysis was done separately for each sample of ocular fluid and plasma. After importing.cel and.arr files, RMA-DABG summarization algorithm was applied using Expression Console with applied “only Homo sapiens” default data analysis settings. The RMA algorithm fits a robust linear model at the probe level to minimize the effect of probe-specific affinity differences. RMA consists of three steps: (1) Background adjustment; (2) Quantile normalization; (3) Summarization This is a multi-chip analysis approach. Therefore, all arrays intended for comparison were included together in the summarization step [8]. The output files of the RMA analysis are.chp files. Differentially expressed miRNAs were identified using Transcriptome Analysis Console (TAC, Affymetrix). One way ANOVA was used to identify statistically significant genes at the significance level of p≤0.05. To identify biologically relevant gene expression changes for each of the time point/treatment conditions, the standard approach was employed using a p-value (p≤0.05) as the primary criterion followed by fold change (-1.5≥ FC ≥1.5) as the secondary criterion to select differentially expressed genes. This approach ensures control of false-positive error and preserves the desired biological significance [9]. Upon first analysis, criteria were relaxed to (-1.2≥ FC ≥1.2; p<0.05) to be able to capture trends of all the family members of miRNAs of interest in all the fluids. The analysis was done for each ocular fluid and plasma samples separately. Differential miRNA expression for each of the groups (DMII, DMI, and DMII-NPDR) was established by comparison against the control group. The data discussed in this publication have been deposited in NCBI's Gene Expression Omnibus [10, 11] are accessible through NCBI GEO Series accession number # GSE140959 (https://www.ncbi.nlm.nih.gov/geo/query/acc.cgi?acc=GSE140959).

### Quantitative polymerase chain reaction (qPCR) assays

Real-time qPCR assays (TaqMan assays, ThermoFisher Scientific) were run on miRNA samples isolated from plasma samples from three patients groups vs. controls, on three replicas for each group and in triplicate for each replica. Assays were run at the Real-time PCR Research and Diagnostics Core Facility at UC Davis. Each miRNA sample was amplified using a TaqMan preamplification procedure and TaqMan preamp primers (Applied Biosystems, Life Technologies, Grand Island, NY) following manufacturers protocols. Reverse transcription thermal-cycling conditions were: 16°C/30 min, 42°C /30 min, 85°C /5 min, hold 4°C /∞. Pre-amplification thermal-cycling conditions were: 95°C /10 min, 55°C /2 min, 72°C /2 min, 95°C /15 s, 12 cycles of 95°C /15 s, 60°C /4 min, hold at 99.9°C /10 min, hold 4°C /∞. The amplified samples were run at a dilution of 1:100, in 384 well plates. A control with no enzyme in the reverse transcriptase (RT) step was included as a negative control. Real-time thermal-cycling conditions: hold 95°C /10 min, 40 cycles of 95°C /15 sec, 60°C /60 s, hold 4°C /∞. The TaqMan assays performed were let-7a (ABI No. 000377, hsa-let-7a), let-7b (ABI No. 002619, hsa-let-7b-5p) and let-7c (ABI No. 000379, hsa let-7c). Control genes were miR-3613 (ABI No. 463197_mat, hsa-miR-3613-5p), miR-4487 (ABI No. 462492_mat, hsa-miR-4487), and miR-638 (ABI No. 001582, hsa-miR-638).

### Pathway analysis

Pathway and gene network analysis of miRNAs and their target genes was performed using Ingenuity Pathway Analysis (IPA, Qiagen, Redwood City, CA). IPA is a web-based software application that enables one to analyze, integrate, and understand the significance of the data, in the context of larger biological systems. IPA is backed by the Ingenuity^®^ Knowledge Database of highly structured, detailed biological findings manually curated by Ph.D. level scientists. MiRNA Target Filter combines filtering tools and miRNA-mRNA content to provide insight into the biological targets of candidate miRNAs.

## Results

### Differentially expressed miRNAs in aqueous, vitreous, and plasma of diabetics patients

Results for each of the three groups of Diabetes mellitus (DM) vs. control and each fluid examined are presented below. Statistical data analysis was done using ANOVA with cut off criteria of (-1.5≥ FC ≥1.5, p<0.05). Only the most interesting differentially expressed miRNA species are presented here, while the full lists of the expressed molecules (p<0.05) are available in the **(S2–S4 Tables)**.

### Differentially expressed miRNAs in aqueous humor

**Fig 1:**
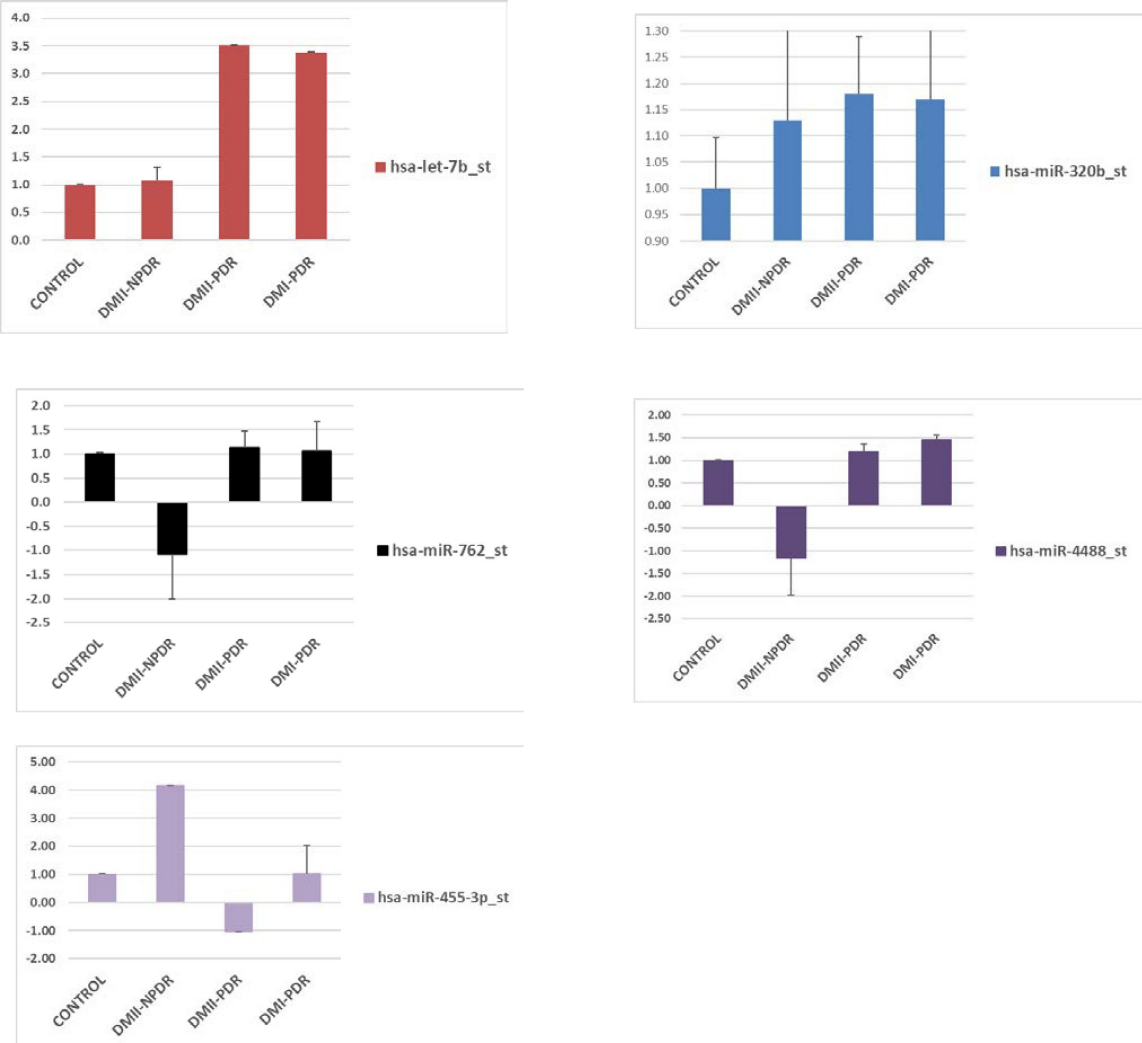
Select set of differentially expressed miRNA in aqueous in DRI-PDR, DRII-PDR and DRII-NPDR.

The miRNAs that have been statistically significantly (p<0.05) differentially expressed in aqueous for each patients sample group compared to controls are presented in **Fig 1** and listed in **Table 1**. The miRNA that has been the most dysregulated in aqueous in DMI-PDR and DMII-PDR is let-7b. In DRII-NPDR, it was miR-455 (**Table 1**). Dysregulated miRNAs that have been found in multiple sub-categories are let-7b (DMI-PDR and DMII-PDR), miR-26a (DMII-PDR and DMII-NPDR); and miR-4314 and miR-518 (DMI-PDR and DMII-PDR) (**Table 1**). Each of the aqueous DR subcategories had a set of 12–35 additional unique differentially expressed miRNAs, for example, miR-3202 (DMI-PDR), miR-296 (DMII-PDR), and miR-455 (DMII-NPDR) to mention just a few (**S2 Table**).

**Table 1:** Differentially expressed miRNA in aqueous in DRI-PDR, DRII-PDR and DRII-NPDR.

No	Transcript Cluster ID	DMII-NPDR (FC)	DMII-NPDR (p-value)	DMII-PDR (FC)	DMII-PDR (p-value)	DMI-PDR (FC)	DMI-PDR (p-value)
**1**	hsa-let-7b_st	1.08	0.232	3.51	0.012^a^	3.38	0.012^a^
**2**	hsa-miR-3665_st	1.44	0.529	1.7	0.225	1.84	0.290
**3**	hsa-miR-2861_st	1.95	0.066	1.32	0.603	1.08	0.682
**4**	hsa-miR-296-3p_st	1.11	0.151	1.28	0.001^b^	1.02	0.796
**5**	hsa-miR-563_st	-1.06	0.533	1.25	0.010^a^	1.07	0.575
**6**	hsa-miR-26a_st	1.35	0.014^b^	1.23	0.027^a^	1.12	0.248
**7**	hsa-miR-4488_st	-1.17	0.814	1.18	0.186	1.46	0.094
**8**	hsa-miR-4466_st	-1.04	0.848	1.16	0.537	1.38	0.580
**9**	hsa-miR-4665-5p_st	1.07	0.517	1.13	0.544	1.3	0.023^a^
**10**	hsa-miR-762_st	-1.09	0.911	1.13	0.341	1.06	0.602
**11**	hsa-miR-3202_st	1.19	0.004^b^	1.1	0.120	1.38	0.008^b^
**12**	hsa-miR-29a_st	1.03	0.633	1.03	0.703	1.31	0.001^b^
**13**	hsa-miR-1267_st	1.07	0.306	1.02	0.876	1.35	0.002^b^
**14**	hsa-miR-122-star_st	1.26	0.011^b^	-1.01	0.386	1.15	0.024^a^
**15**	hsa-miR-204_st	1.36	0.026	-1.03	0.843	-1.04	0.739
**16**	hsa-miR-455-3p_st	4.15	0.002^b^	-1.05	0.624	1.03	0.993
**17**	hsa-miR-548al_st	-1.22	0.004^b^	-1.06	0.156	-1.13	0.047^a^
**18**	hsa-miR-4314_st	-1.02	0.915	-1.24	0.047^a^	-1.23	0.038^a^
**19**	hsa-miR-518c_st	-1.04	0.229	-1.29	0.010^b^	-1.25	0.046^a^

FC = Fold Change

^a^p ≤ 0.05

^b^p ≤ 0.01

### Differentially expressed miRNAs in the vitreous humor

**Fig 2:**
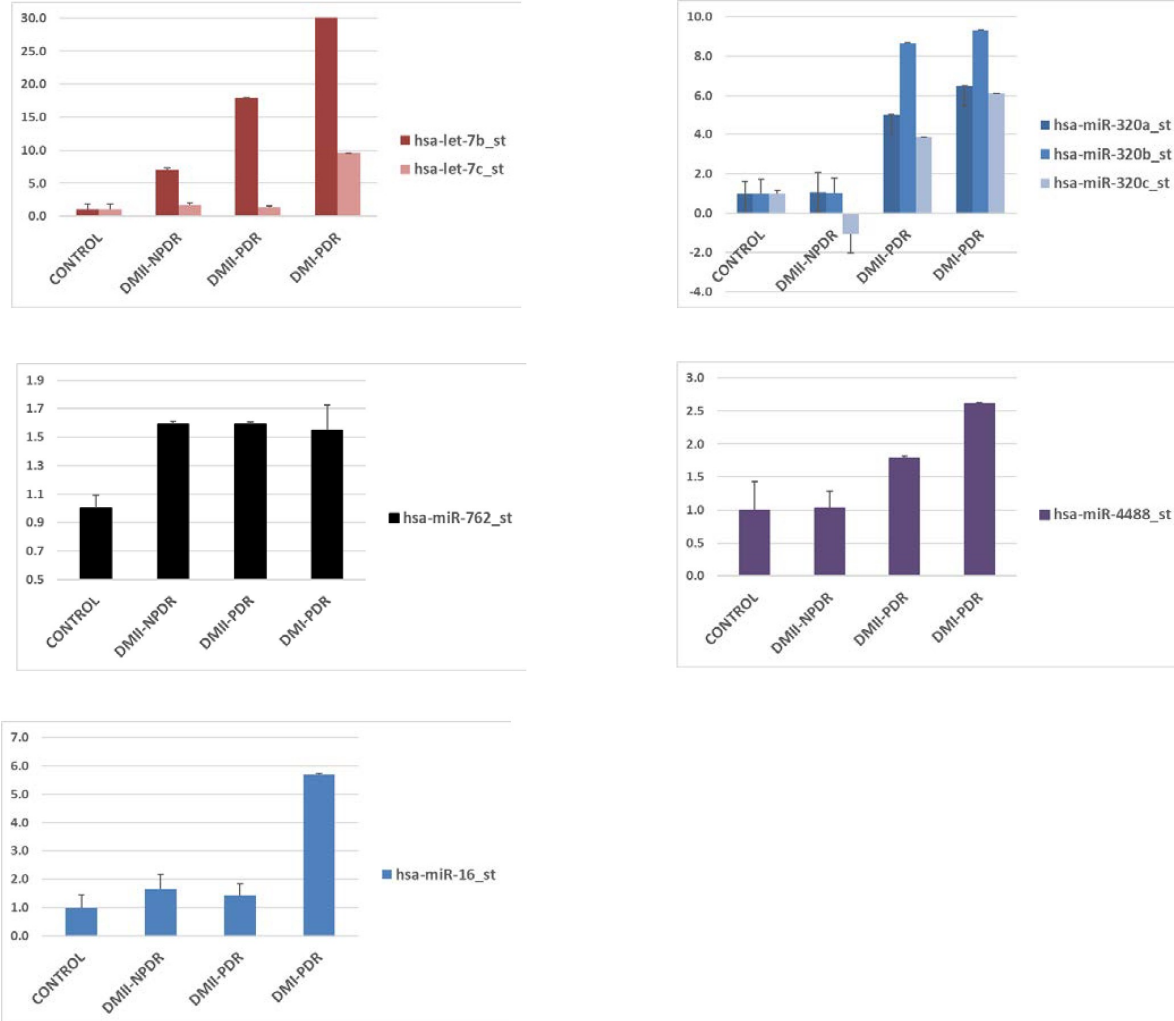
Select set of differentially expressed miRNA in vitreous in DRI-PDR, DRII-PDR and DRII-NPDR.

The miRNAs that have been statistically significantly (p<0.05) differentially expressed in vitreous for each patients sample group compared to controls are presented in **Fig 2** and listed in **Table 2**. The miRNA that has been the most dysregulated in vitreous of all three categories was let-7b. In DMI-PDR and DMII-PDR, it was miR-320b and miR-320c, and in DMII-NPDR, it was miR-2861 (**Fig 2**). MiR-4488 was dysregulated in two subcategories, DMI-PDR and DMII-PDR. Dysregulated miRNAs that have been found in all three DM subcategories was miR-762 (**Table 2**). Each of the vitreous subcategories had a set of additional 15–46 unique differentially expressed miRNAs that were present in only one of the subcategories, for example, miR-486-5p and miR-16(DMI-PDR), miR-4668 (DMII-PDR), miR-2861 (DMII-NPDR) (**S3 Table**).

**Table 2:** Differentially expressed miRNA in vitreous in DRI-PDR, DRII-PDR and DRII-NPDR.

No	Transcript Cluster ID	DMII-NPDR (FC)	DMII-NPDR (p-value)	DMII-PDR (FC)	DMII-PDR (p-value)	DMI-PDR (FC)	DMI-PDR (p-value)
**1**	hsa-let-7b_st	6.94	0.388	17.92	0.065	58.25	0.015^a^
**2**	hsa-let-7c_st	1.66	0.367	1.39	0.137	9.57	0.009^b^
**3**	hsa-let-7d_st	1.26	0.461	1.29	0.521	1.67	0.023^a^
**4**	hsa-miR-486-5p_st	-1.03	0.357	1.39	0.154	9.43	0.020^a^
**5**	hsa-miR-320a_st	1.07	0.983	5	0.060	6.49	0.018^a^
**6**	hsa-miR-320b_st	1.02	0.772	8.64	0.054^a^	9.32	0.043^a^
**7**	hsa-miR-320c_st	-1.05	-0.986	3.86	0.025^a^	6.12	0.011^b^
**8**	hsa-miR-92a_st	1.01	0.468	1.81	0.089	7.71	0.017^a^
**9**	hsa-miR-185_st	1.02	0.847	1.28	0.193	5.91	0.007^b^
**10**	hsa-miR-16_st	1.65	0.519	1.42	0.411	5.68	0.049^a^
**11**	hsa-miR-4488_st	1.03	0.261	1.79	0.025^a^	2.61	0.018^a^
**12**	hsa-miR-4695-5p_st	1.15	0.253	1.75	0.000^b^	1.76	0.005^b^
**13**	hsa-miR-762_st	1.59	0.021^a^	1.59	0.014^a^	1.55	0.176
**14**	hsa-miR-4421_st	1.23	0.001^b^	1.13	0.085	1.3	0.000^b^
**15**	hsa-miR-3688-3p_st	1.35	0.025^a^	1.14	0.226	1.25	0.047^a^
**16**	hsa-miR-4683_st	1.18	0.074	1.17	0.061	1.25	0.034^a^
**17**	hsa-miR-512-3p_st	1.1	0.016^a^	1.27	0.016^a^	1.23	0.019^a^
**18**	hsa-miR-4668-3p_st	-1.1	0.752	1.25	0.004^b^	-1.06	0.849
**19**	hsa-miR-3201_st	-1.43	0.640	-3.83	0.005^a^	-4.77	0.004^b^

^a^p ≤ 0.05

^b^p ≤ 0.01

### Differentially expressed miRNAs in plasma

**Fig 3:**
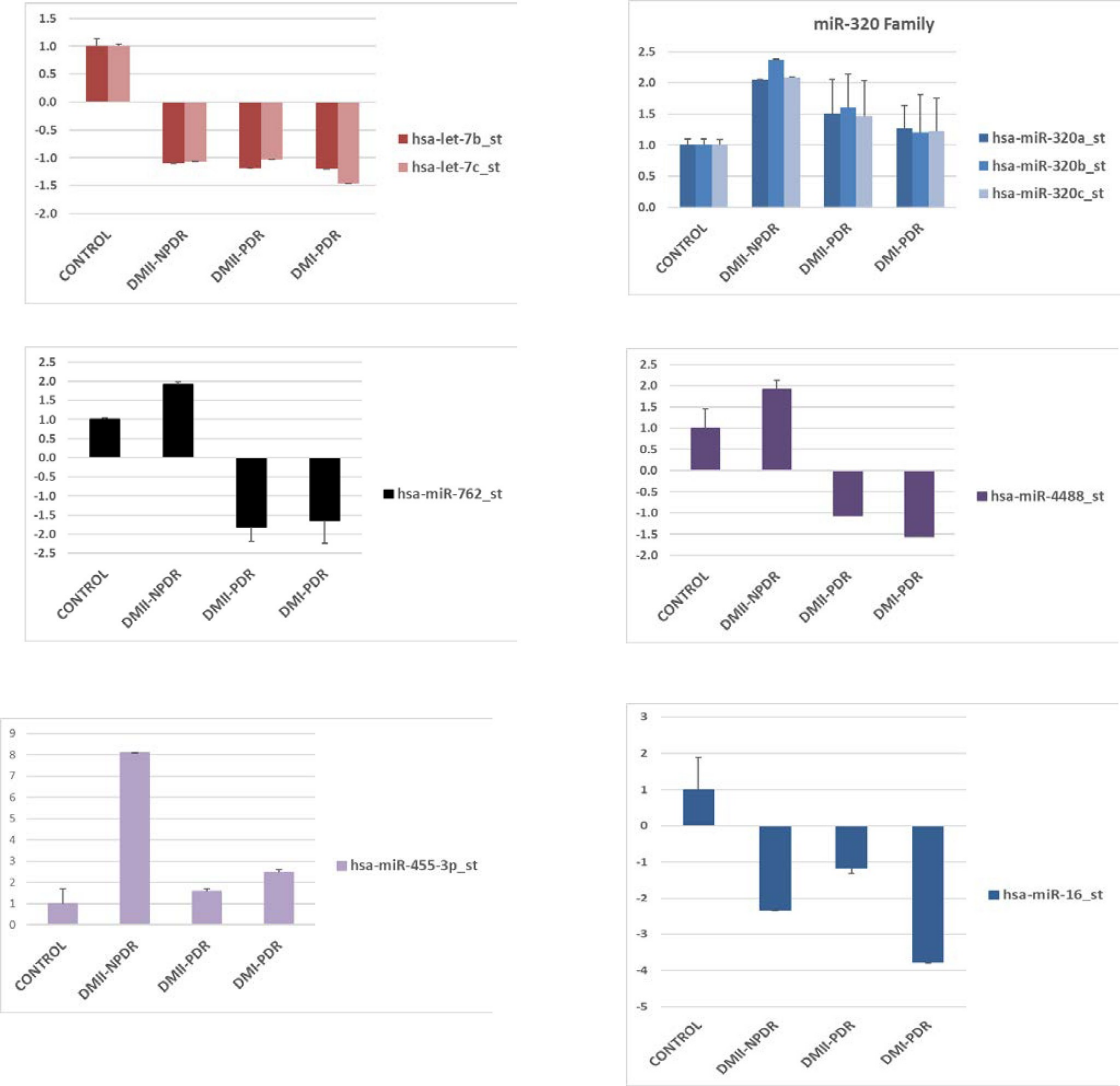
Select set of differentially expressed miRNA in plasma in DRI-PDR, DRII-PDR and DRII-NPDR.

The miRNAs that have been statistically significantly (p<0.05) differentially expressed in plasma for each patients sample group compared to controls are presented in **Fig 3** and listed in **Table 3**. The miRNA that had been the most dysregulated in plasma in DMI-PDR was miR-106b, in DMII-PDR, it was miR-20b, while in DRII-NPDR, it was miR-20b (**Fig 3**). Dysregulated miRNAs that have been found in 2 subcategories were miR-455, miR-20a, and miR-20b (DMI-PDR and DMII-PDR). Additionally, there were 13 miRNAs that seemed to be common biomarkers for DM (**S4 Table**). Each of the plasma subcategories had a set of additional 5–42 unique differentially expressed miRNAs that are present in only one of the subcategories, for example, miR-574-3p (DMI-PDR), miR-4695-5p (DMII-PDR), and miR-455-3p (DMII-NPDR) (**S4 Table**).

**Table 3:** Differentially expressed miRNA in plasma in DRI-PDR, DRII-PDR and DRII-NPDR.

No	Transcript Cluster ID	DMII-NPDR (FC)	DMII-NPDR (p-value)	DMII-PDR (FC)	DMII-PDR (p-value)	DMI-PDR (FC)	DMI-PDR (p-value)
**1**	hsa-let-7a_st	-2.14	0.114	-2	0.088	-3.82	0.085
**2**	hsa-let-7e_st	-2.08	0.825	-1.9	0.352	-2.33	0.894
**3**	hsa-let-7g_st	-9.32	0.028^a^	-3.24	0.152	-11.09	0.171
**4**	hsa-let-7i_st	-3.22	0.020^a^	-1.03	0.113	-4.53	0.031^a^
**5**	hsa-miR-106b-star_st	-10.6	0.013^b^	-2.95	0.249	-10.32	0.029^a^
**6**	hsa-miR-126_st	-4.74	0.061	-1.8	0.127	-6.94	0.067
**7**	hsa-miR-143_st	-3.84	0.094	-1.44	0.333	-3.7	0.192
**8**	hsa-miR-150_st	-4.66	0.039^a^	-1.71	0.281	-3.36	0.293
**9**	hsa-miR-151-5p_st	-1.12	0.510	-1.59	0.140	2.18	0.266
**10**	hsa-miR-16_st	-2.33	0.008^b^	-1.17	0.139	-3.78	0.029^a^
**11**	hsa-miR-18a_st	-11.31	0.016^a^	-3.45	0.117	-6.35	0.054^a^
**12**	hsa-miR-18b_st	-2.38	0.048^a^	-2.02	0.238	-2.13	0.281
**13**	hsa-miR-192_st	-4.28	0.013^b^	-3.51	0.146	-4.65	0.050^a^
**14**	hsa-miR-194_st	-8.32	0.007^b^	-2.52	0.312	-10.2	0.014^b^
**15**	hsa-miR-199b-3p_st	-1.62	0.909	-2.41	0.664	-1.64	0.884
**16**	hsa-miR-20a_st	-3.81	0.000^b^	-2.05	0.044^a^	-2.81	0.009^b^
**17**	hsa-miR-20b_st	-39.65	0.000^b^	-8.06	0.005^b^	-12.82	0.003^b^
**18**	hsa-miR-221_st	-5.03	0.180	2.33	0.577	2.73	0.421
**19**	hsa-miR-26a_st	-2.2	0.146	-2.8	0.067	-2.98	0.123
**20**	hsa-miR-27a_st	-4.52	0.040^a^	-2.14	0.314	-12.21	0.060
**21**	hsa-miR-29a_st	-6.37	0.057	-1.36	0.456	-4.7	0.070
**22**	hsa-miR-30c_st	-2.16	0.264	-1.33	0.944	-2.08	0.828
**23**	hsa-miR-320a_st	2.05	0.006^b^	1.51	0.543	1.27	0.365
**24**	hsa-miR-320b_st	2.37	0.005^b^	1.6	0.540	1.2	0.609
**25**	hsa-miR-320c_st	2.09	0.009^b^	1.47	0.559	1.22	0.526
**26**	hsa-miR-342-3p_st	-4.03	0.207	-3.27	0.150	-4.73	0.154
**27**	hsa-miR-363_st	-8.42	0.012^b^	-1.87	0.471	-9.56	0.061
**28**	hsa-miR-455-3p_st	8.09	0.020^a^	1.58	0.113	2.48	0.104
**29**	hsa-miR-4668-5p_st	1.06	0.847	2.12	0.175	3.14	0.352
**30**	hsa-miR-486-3p_st	-2.98	0.041^a^	-2.38	0.217	-3.45	0.135
**31**	hsa-miR-500a-star_st	-2.43	0.041^a^	-1.34	0.481	-2.87	0.106
**32**	hsa-miR-502-3p_st	-2.68	0.040^a^	-1.38	0.485	-4.04	0.123
**33**	hsa-miR-532-5p_st	-4.85	0.051^a^	-1.78	0.406	-5.5	0.045^a^
**34**	hsa-miR-762_st	1.92	0.071	-1.81	0.384	-1.64	0.598

^a^p ≤ 0.05

^b^p ≤ 0.01

### Comparison of differentially expressed miRNAs in all three fluids

**Fig 4:**
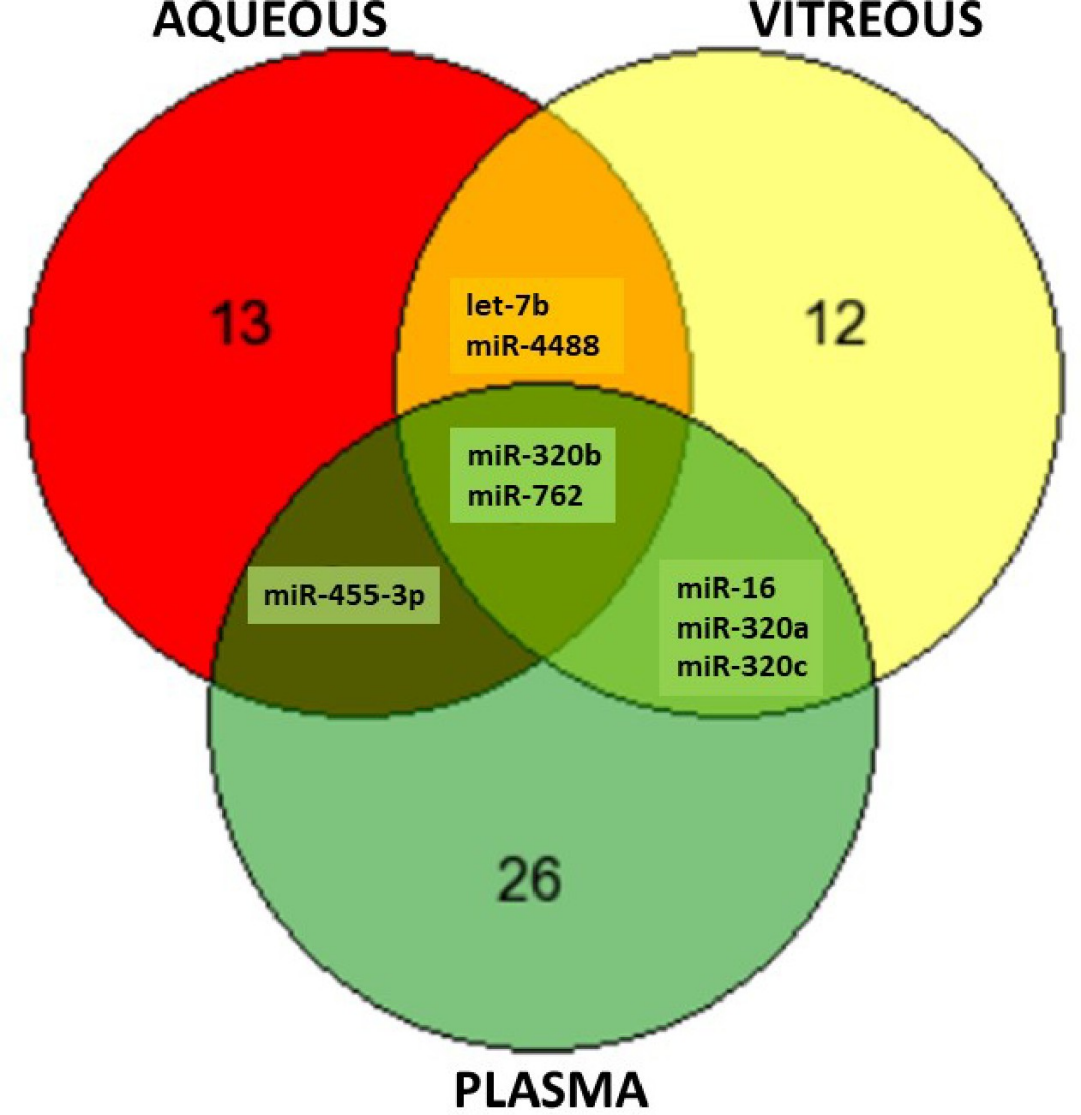
Summary venn diagram of the most promising microRNA biomarkers.

When compared aqueous, vitreous, and plasma for all DR category, four miRNAs have been found dysregulated in all three compartments. Those miRNAs were let-7b, miR-320b, mir-762, and miR-4488. MiRNA let-7b was upregulated in aqueous and vitreous, while downregulated in plasma. MiR-320b was upregulated in all three fluidics compartments. MiR-762 and miR-4488 follow a similar pattern of expression–they have a category-specific expression in aqueous, upregulated in vitreous and category-specific expression in plasma (**Fig 4**).

### Comparative analysis of miRNA dysregulated in aqueous, vitreous and plasma in each DM category

**Fig 5:**
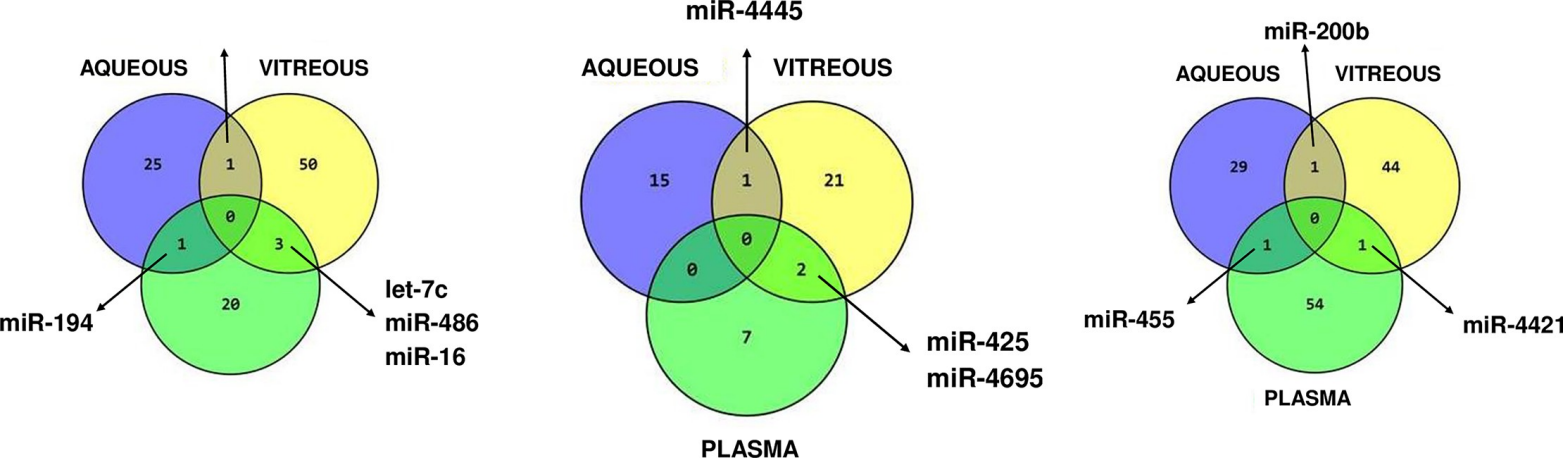
The comparative analyses of miRNA dysregulation in aqueous, vitreous, and plasma for each DR sub-category. **A.** DMI-PDR; **B.** DMII-PDR; **C.** DMII-NPDR.

Several miRNAs have been identified that are statistically significantly (p<0.05) dysregulated in multiple fluidic compartments. The comparative analyses of miRNA dysregulation in aqueous, vitreous, and plasma for each DR sub-category are presented in **Fig 4** and **Table 4A–4C**. When compared aqueous, vitreous, and plasma for DMI-PDR category (p<0.05), dysregulation of let-7b was present in aqueous and vitreous, while dysregulation of miR-194 was present in aqueous and plasma. The vitreous and plasma shared statistically significant dysregulation of 3 miRNA: let-7c, miR-486, and miR-16. Interestingly, miRNA candidate biomarkers were upregulated in ocular fluids and downregulated in plasma (**Fig 5A and Table 4A**). In **S5 Table** are listed some of the unique miRNAs for each fluid. The most dysregulated unique miRNA for aqueous was miR-3202, for vitreous miR-320b and plasma miR-15a (**S5 Table**). When compared aqueous, vitreous, and plasma for DMII-PDR category (p<0.05), downregulation of miR-4445 was present in aqueous and vitreous, while upregulation of miR-4695-5p and miR-425 was present in vitreous and plasma (**Fig 5B** and **Table 4B**). On the bottom panel of **S6 Table** are listed some of the unique miRNAs for each fluid. The most upregulated unique miRNA for aqueous was let-7b, for vitreous miR-320c and in plasma, it was downregulated miR-20b (**S6 Table**). When compared aqueous, vitreous and plasma for DMII-NPDR category (p<0.05), upregulation of miR-455-3p was present in aqueous and plasma, upregulation of miR-200b was shared by aqueous and vitreous, while upregulation of miR-4421 was present in vitreous and plasma (**Fig 5C** and **Table 4C**). On the bottom panel of **S7 Table** are listed some of the unique miRNAs for each fluid. The most dysregulated unique miRNA for aqueous was miR-3201, for vitreous miR-2861, and for plasma, it was downregulated miR-20b (**S7 Table**).

**Table 4:** Candidate miRNA biomarkers overlapping in different fluids.

**A. DMI-PDR**						
**miRNA**	**AQC-DMI-PDR**	**p-value**	**VIT-DMI-PDR**	**p-value**	**PLS-DMI-PDR**	**p-value**
**hsa-let-7c_st**	1.35	0.217	9.57	0.009^b^	-1.46	0.036^a^
**hsa-miR-486-5p_st**	1.05	0.763	9.43	0.200	-1.62	0.017^a^
**hsa-miR-16_st**	1.06	0.559	5.68	0.049^a^	-3.78	0.029^a^
**hsa-miR-194_st**	1.2	0.011^b^	-1.01	0.816	-10.2	0.014^b^
**hsa-let-7b_st**	3.38	0.012^b^	58.25	0.015^a^	-1.2	0.240
**hsa-miR-320b_st**	1.17	0.260	9.32	0.043^a^	1.2	0.609
**hsa-miR-762_st**	1.06	0.602	1.55	0.176	-1.64	0.598
**hsa-miR-4488_st**	1.46	0.094^c^	2.61	0.018^a^	-1.56	0.614
**B. DMII-PDR**						
**miRNA**	**AQC-DMII-PDR**	**p-value**	**VIT-DMII-PDR**	**p-value**	**PLS-DMII-PDR**	**p-value**
**has-miR-4445-star_st**	-1.22	0.025^a^	-1.41	0.044^a^	1.11	0.464
**has-miR-4695-5p_st**	1.08	0.459	1.75	0.000^b^	1.67	0.036^a^
**has-miR-425-star_st**	1.02	0.900	1.21	0.013^b^	1.44	0.006^b^
**has-let-7b_st**	3.51	0.012^a^	17.92	0.065^c^	-1.19	0.167
**has-miR-320b_st**	1.18	0.109	8.64	0.054^a^	1.6	0.540
**has-miR-762_st**	1.13	0.341	1.59	0.014^a^	-1.81	0.384
**has-miR-4488_st**	1.18	0.186	1.79	0.025^a^	-1.07	0.465
**C. DMII-NPDR**						
**miRNA**	**AQC-DMII-NPDR**	**p-value**	**VIT-DMII-NPDR**	**p-value**	**PLS-DMII-NPDR**	**p-value**
**hsa-miR-455-3p_st**	4.15	0.002^b^	3.06	0.166	8.09	0.020^a^
**hsa-miR-4421_st**	1.02	0.853	1.23	0.001^b^	1.24	0.009^b^
**hsa-miR-200b-star_st**	1.23	0.035^a^	1.21	0.032^a^	1.12	0.037^a^
**hsa-let-7b**	1.08	0.232	6.94	0.388	-1.1	0.751
**hsa-miR-320b_st**	1.13	0.287	1.02	0.772	2.37	0.005^b^
**hsa-miR-762_st**	-1.09	0.911	1.59	0.021^a^	1.92	0.071^c^
**hsa-miR-4488_st**	-1.17	0.814	1.03	0.261	1.91	0.219

^a^p ≤ 0.05

^b^p ≤ 0.01

^c^p close to 0.05

### Validation of microarray results with qPCR

Independent quantitative PCR (qPCR) validation of representative differentially expressed genes was performed using commercial gene expression assays (TaqMan; Applied Biosystems), following manufacturers protocol (for detailed reaction conditions see Materials and Methods section on Quantitative polymerase chain reaction (qPCR) assays). The goal of the qPCR experiment was to validate microarray results by alternative technique, and the criteria to pick samples was a random pick within the DR groups. The fold changes (FC) and their direction were confirmed for the chosen sets of genes. Data are presented in **Table 5**.

**Table 5:** Comparison of microarray results with qPCR.

PLASMA	miRNA	microarray Fold Change	p-value (ANOVA)	qPCR Fold Change	p-value (t-test)
**DMI-PDR vs con**	**hsa-let-7a**	-3.82	0.08	-1.96	0.58
	**has-let-7b**	-1.2	0.24	-1.34	0.42
	**hsa-let-7c**	-1.46	0.04	-2.22	0.22
**DMII-PDR vs con**	**hsa-let-7a**	-2	0.09	-4.07	0.10
	**has-let-7b**	-1.19	0.17	-5.38	0.005
	**hsa-let-7c**	-1.03	0.17	-7.67	0.003
**DMII-NPDR vs con**	**hsa-let-7a**	-2.14	0.11	-12.37	0.07
	**has-let-7b**	-1.1	0.75	-13.22	0.013
	**hsa-let-7c**	-1.06	0.40	-20.66	0.02

### IPA pathway analysis

**Fig 6:**
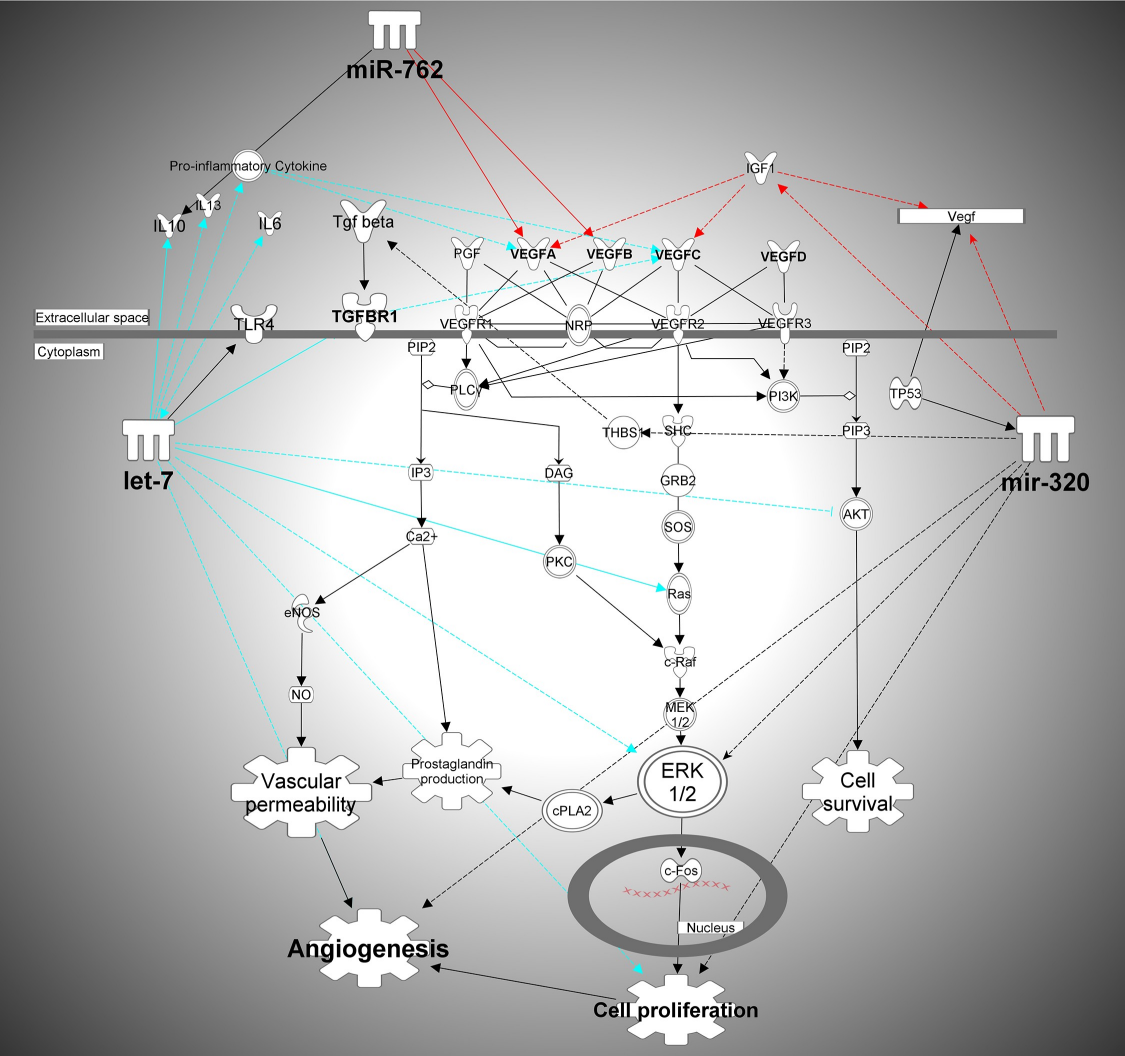
IPA analysis of pathways targeted by both let-7 and miR-320 family as identified pathways connecting VEGF and TGF-beta.

Top Gene Network affected by ubiquitously expressed miRNA in aqueous and vitreous Ingenuity Pathways Analysis was done using 30 highest level expressing miRNAs from aqueous and vitreous. Circulating miRNAs that are ubiquitously present in ocular chambers target primarily genes regulated by p53 and TGF-beta. A partial interactome of the let-7 family of miRNAs and some of the target genes and biological pathways that they regulate are TGF-β, Insulin Receptor, Apoptosis, and VEGF Receptor Signaling. These genes are key regulators of oxidative stress, angiogenesis, inflammation, and apoptosis. MiR-320 family is as part of the cellular response to glucose stimulus and plays a role in apoptosis, migration, cell death, proliferation, and signaling in several diseases including non-insulin-dependent diabetes mellitus (IPA and refs therein). According to IPA summary, miR-320 family regulates a multitude of genes, including IGF1, and TGF beta, and it is regulated by p53 and Smad2/3, which is downstream from TGF-beta. Further IPA analysis of pathways targeted by both let-7 and miR-320 family have identified pathways connecting VEGF and TGF-beta, as represented in **Fig 6**.

## Discussion

One of the primary goals of this project was to make progress towards generating biomarker profiles that can be used to screen for DM stage and progression of DR. Once biomarkers have been identified, the role of these microRNAs as therapeutic targets could be studied.

This pilot study showed dysregulation of many miRNAs, but just a few displayed dysregulations in multiple fluids. One such potential biomarker is the let-7 family of miRNAs. This family was upregulated in the aqueous and vitreous humor and downregulated in plasma of DMI-PDR. The let-7 family also showed dysregulation in more than one category of DR. It was upregulated in vitreous and downregulated in plasma of DMII-NPDR, and upregulated in aqueous, vitreous and downregulated in plasma of DMII-PDR and DMI-PDR. In aqueous upregulation occurred only for PDR categories, so in this compartment, let-7b has the potential to be a biomarker of PDR. The pathway analysis of let-7 targets has shown that let-7 targets proinflammatory cytokines, which regulate VEGF expression. It has been shown that let-7 family members are direct translational repressors of interleukin-13. Jiang et al. have been shown that serum levels, as well as IL-13, secreted from cultured skeletal muscle, are reduced in T2DM vs. normal glucose-tolerant (NGT) subjects, while let-7 is increased [12].

Additionally, the polymorphism in the let-7 targeted region of the Lin28 gene, which codes for long noncoding (lncRNA) a negative regulator of let-7, is associated with an increased risk of type 2 diabetes mellitus [13]. Upregulation of let-7 in ocular fluids might be a sign of nerve damage since it has been shown that the upregulation of let-7 in the extracellular space can lead to neurodegeneration [14]. In one study, the RNA sensing receptor Toll-like7 (TLR7) in cortical neurons of mice was shown to bind extracellular enriched *let-7* released by degenerating neurons. Subsequently, the TLR 7 expressing cells undergo apoptosis. Injection of *let-7b* was also sufficient to activate downstream TLR7 signaling, which was shown by the increased phosphorylation state of IRAK4 [15].

Let-7 has also been implicated in post-transcriptional control of the innate immune response. Macrophages stimulated with live antigens downregulate the expression of several members of let-7 miRNA to relieve repression of immune-modulatory cytokine IL-6 and IL-10 [16, 17]. Let-7 has been implicated in the negative regulation of TLR4, the major immune receptor of microbial lipopolysaccharide (LPS), and down-regulation of let-7 both upon microbial and protozoan infection might elevate TLR4 signaling and expression [18, 19]. Let-7 is also a very attractive potential therapeutic that can prevent tumorigenesis and angiogenesis, so far in cancers, and possibly in DR [20].

Another family of miRNA, miR-320, appears in multiple fluids and DR categories. miR-320 family is upregulated in vitreous of both DRI-PDR and DRII-PDR, therefore it might be considered as a putative vitreous biomarker of PDR. MiR-320 regulates tumor angiogenesis driven by vascular endothelial cells in oral cancer by silencing neuropilin 1 [21]. Neuropilin 1 functions as a co-receptor with diverse ligands and receptors, including the vascular endothelial growth factor (VEGF) and VEGF-receptor (VEGFR). Wang et al. have shown that in type 2 diabetic Goto-Kakizaki (GK) rats myocardial microvascular endothelial cells (MMVEC) miR-320 impaired angiogenesis [22] and that one of the miR-320 targets is IGF-1. Eleven miRNAs were upregulated in MMVEC from GK rats compared with those in Wistar rats including let-7e, and miR-320. The results indicate that the upregulation of miR-320 in MMVEC from GK rats may be responsible for the inconsistency between the expression of IGF-1 protein and mRNA and therefore related to impaired angiogenesis in diabetes. Transfection of a miR-320 inhibitor was suggested as a therapeutic approach for the treatment of impaired angiogenesis in diabetes [22]. This miRNA was also found upregulated in insulin-resistant 3T3-L1 adipocytes. Anti-miR-320 oligo was found to regulate insulin resistance in adipocytes by improving insulin–PI3-K signaling pathways [23]. MiR-320 has been found to regulate glucose-induced gene expression in Diabetes [24]. High glucose exposure decreased the expression of miRNA 320 (miR-320) but increased the expression of endothelin 1 (ET-1), vascular endothelial growth factor (VEGF), and fibronectin (FN) in human umbilical vein endothelial cells (HUVECs). Data from this study indicate that miR-320 negatively regulates expression of ET-1, VEGF, and FN through ERK 1/2 in HUVECs. Increased expression of miR-320 family in our data could suggest that ocular tissue is attempting to downregulate a high level of VEGF production, which has been shown to occur in the diabetic eye.

One of the miRNAs that have shown up as a putative candidate biomarker in all three fluids is miR-762. Downregulation of this miRNA has been associated with increased plasma VEGF levels following ischemic preconditioning, and algorithm-based database searches suggested that this miRNA bind to the 3' UTR of VEGF mRNA, which was confirmed with *in vitro* knockdown of miRNA expression experiments in CD34-positive BM cells [25]. MiR-762 has also been identified as having the potential neuroprotective role in neurorestorative therapy for ischemic stroke [26].

The fourth potential biomarker miRNA is miR-4488. Microarray profiling of the overexpression of TGFβ2-OT1 indicates that miR-4488 is one of the three miRNAs whose overexpression in endothelial cells resulted in the repression of their downstream targets: ceramide synthase 1 (CERS1), N-acetyltransferase 8-like (NAT8L), and La-ribonucleoprotein-domain-family-member 1 (LARP1). Their primary function is in endothelial cell autophagy, inflammation in endothelial injury, and regulation of angiogenesis [27].

According to IPA analysis, one of the main pathways that have been targeted by miRNAs in ocular fluids are VEGF and TGF-beta pathways. TGF-beta has an important role in angiogenesis, endothelial cell proliferation, adhesion, and deposition of extracellular matrix [28, 29]. TGF-beta has been implicated in the development of diabetic retinopathy (DR) through disrupting angiogenesis and blood-retinal barrier breakdown [30]. TGF-beta is a highly polymorphic gene, and systematic review has been published that has evaluated TGF-beta1 gene polymorphism in association with Diabetic retinopathy susceptibility, which suggested that +869T/C(L10P) polymorphism in TGF-beta1 gene would be a potential protective factor for DR [31]. It is interesting to hypothesize that perhaps a polymorphism like that could be a target site of a particular miRNA that gets dysregulated in diabetic patient, and if a protective allele is present, complementarity of the binding site is disrupted and there is no effect on TGF-beta1 expression, as it would be if sensitive allele is present.

There are several miRNAs dysregulated in only one of the fluids that might be potential biomarkers for PDR. In aqueous, two miRNAs were downregulated miR-4314 and miR-518c in both DRI-PDR and DRII-PDR. Serum miRNA-4314 has been implicated in ovarian tumorigenesis via down-regulating GRWD1/IP6K1/NEGR1 [32]. MiRNA-518c, together with miR-638, are dual PTEN- and p53-targeting miRNAs that are upregulated in multiple human cancers [33]. MiR-518 also plays a role in the growth and metastasis of several cancers, where it has been identified as a downstream target of the SDF-1/CXCR4 system. It was also found in a cluster of miRNAs that are highly expressed in retinoblastoma [34]. In vitreous, there was upregulation of miR-320c, miR-4488, miR-4695, miR-512-3p and downregulation of miR-3201. In plasma, the upregulation of miR-425 might be a biomarker for PDR. MiRNA-425 has been already considered as therapeutic targets and biomarkers of cardiovascular disease because it binds to a polymorphic region of 3’UTR of its target atrial natriuretic peptide (ANP) mRNA. This A/G variant is contained within a binding site for miR-425, which binds to the A but not the G allele, and ANP expression is elevated in individuals with the G allele, correlating with reduced blood pressure (functional polymorphism). These findings raise the possibility that inhibitors of miR-425 might lower blood pressure by de-repressing atrial natriuretic peptide expression [35]. As far as unique miRNA candidate biomarkers go, upregulation of miR-4695-5p and downregulation of miR-569 was detected in DMII-PDR, and upregulation of miR-574-3p, miR-2115 and miR-28-3p, as well as downregulation of let-7c, miR-107, miR-532-5p and miR-222 were detected in DMI-PDR. MiR-4695-5p has been identified as one of the miRNA targeting TGF beta pathway genes, specifically TGFBR1 (rs6478974) and SMAD3 (rs12901071). The TGF-β signaling pathway is involved in the regulation of cell growth, angiogenesis, and metastasis. The authors have shown from a large study of colorectal cancer cases that genetic variation in the TGF-β signaling pathway is associated with various miRNA expression levels [36]. MiRNA-569 has been associated with a functional polymorphism in the 3'-untranslated region of SPI1 with systemic lupus erythematosus. The findings indicate that an SNP in the 3'-UTR of SPI1 is associated with elevated SPI1 mRNA level and with susceptibility to SLE. Transfection experiments demonstrated that miR-569 inhibits expression of a reporter construct with the 3'-UTR sequence containing the nonrisk allele but not the risk allele [37]. These and many more candidates are listed in the Tables and Appendixes. Our future research direction will be following up and confirming the candidate DR biomarkers on a larger number of individuals.

The limitations of our pilot study were that samples were taken at the surgery point. The decision to do that was guided by the impossibility to get vitreous samples in the clinic. Also, the amount of fluid needed to obtain a sufficient amount of miRNA in discovery study such as this was only obtainable from the surgery. Only the samples from the eyes with no previous surgery were taken into account, and the criterion applied was that no other treatments, such as anti-VEGF or steroid injections, were done at least a year before surgery. Another concern was a possible confounding effect of the PRP laser treatment. Therefore, we compared vitreous samples of DR Type II, PDR with and without PRP laser done, and we found that the history of PRP of retinal tissue was not a source of significant variability of miRNAs in vitreous at the collection time point. Another limitation of our study is that the number of patients in each group is rather small. As this was a pilot experiment, there was no previous information to calculate the minimal sample size. Similarly, before the experiment, we had no observations to check the distribution, so it seemed reasonable to make an assumption that the distribution of the pre-processed data is normal and hence two-sample t-tests and ANOVA are applicable. The same assumption is also made by other proposed methods to calculate sample size [38–41]. Liu and Hwang [42] describe a method for a quick sample size calculation for microarray experiments while controlling FDR, which we followed. We have conducted power analysis [43] to determine the minimal sample size needed to produce statistically significant data, based on the vitreous data for the DMI-PDR group. The recommended sample size was n = 7, or 2n = 14. Our total sample size for this study was 2n = 15, but for some of the groups in this pilot study e.g. DMI and DMII-NPDR groups had only 4 samples for aqueous and vitreous. Therefore, the limitation of this study is small sample size; the conclusion drawn from this study should be further validated in future studies.

Most of the miRNAs in this pilot study had a very moderate change of expression in patient samples. This can be understood in the light of miRNAs functioning as a catalyst, similar to an enzyme, so even the slightest change in their expression can have dramatic effects on the target gene expression levels and many different cellular functions, such as vascular remodeling and angiogenesis [44]. While the majority of miRNAs were upregulated in ocular fluids, the majority of miRNAs were downregulated in plasma. It is possible to hypothesize that there is a re-organization of miRNA prevalence in bodily fluids taking place in disease, but it is too early to speculate a possible mechanism of whole-body level regulation of circulatory miRNA.

There was an increase in studies in recent years identifying miRNAs from human samples as DR biomarkers [45–48]. Since our study was a small scale study, we wanted to compare/contrast with some other studies, to see whether our pilot study brought out same candidates, or different sets. There are some plasma biomarkers common between those studies and this pilot study (let-7a, miR-126, miR-320a, miR-27, miR-126, miR-29, miR-150, miR-30, miR-221) [48], and refs therein. For example, this pilot study has identified miRNA-221 as a biomarker in plasma, downregulated in NPDR (FC = -5.03) and upregulated in DMI-PDR (FC = 2.73) and DMII-PDR (FC = 2.33). Same miRNA has been identified in serum as a biomarker for DR in DMII-PDR by Liu et al, 2018 [49]. It was increased in serum, together with Ang II and VEGF [49]. Finding from this work in plasma of let-7a and miR-151 as potential biomarkers have been confirmed by RNA seq in serum for late-stage and early-stage DMII-DR [47]. The lower number of studies of miRNAs in vitreous of human DR patients found common biomarkers with our pilot study such as let-7c, miR-16, miR-92a, and miR-320a,b [48], and refs therein. Some of the miRNA candidates identified in other studies are from the same families (let-7, miR-320) although not exactly the same member. It is very encouraging to see that our findings have been confirmed by different groups using different techniques. However, there are studies that identify completely different sets of biomarkers, such as RNA-seq study of non-proliferative DR biomarkers for DRII patients in Chinese Han ancestry in serum [46]. This kind of result poses questions of the variability between ethnic groups and redundancy of the miRNA family member function. Therefore, more research is needed, and perhaps a variety of ethnic groups should be studied to address those questions.

The strength of this pilot study is in the fact that the majority of samples are from the three different body compartments from the same set of patients. Therefore the correlations in biomarkers between the aqueous, vitreous and plasma compartments are more likely to be meaningful. The high relative stability of miRNA in clinical tissues and biofluids (e.g. plasma, serum, urine, saliva, etc.) and the ability of miRNA expression profiles to accurately classify discrete tissue types and disease states have positioned miRNA quantification as an up-and-coming new tool for a wide range of diagnostic applications [50–52].

The profiling of circulatory miRNAs identified four putative biomarkers let7b, miR320b, miR-4488, and miR-762 that have dysregulated expression in all three examined fluids, aqueous, vitreous and plasma at the onset of DM or DR. The biomarkers identified in aqueous and vitreous fluids are also differentially regulated in plasma. Several circulatory miRNAs have shown differential presence in normals vs. diabetic retinopathy fluid samples, offering promise for further study for diagnostic or therapeutic purposes. Of additional interest and clinical importance is that a high percentage of patients with eye diseases such as DR develop Alzheimer’s disease (AD). Patients with recent DR (diagnosed within 0–5 years) and established DR (> 5 years) were found to be at a higher risk of AD by 67% and 50% compared to those without DR [53]. Identifying ophthalmic diseases by measurement of suitable biomarkers would enable better screening and treatment of those individuals at risk of AD [53, 54].

As a future goal, we will validate the diagnostic validity of these biomarkers in plasma and aqueous humor on a more significant number of individuals. One of our goals in the follow-up study with a much higher number of samples is to have a separate biomarker discovery and confirmation groups. Also, we will conduct longitudinal studies by obtaining patient samples in the clinic settings at the earlier stages of the disease to determine the predictive value for DR progression and responses to treatment. Our results are the promising beginning of developing a fingerprint of biomarkers that might be able to be used as prognostic markers for PDR development in bodily fluids. Information discovered through these studies may lead to major advances in therapeutic management.

## Supporting information

S1 Table De-identified patient list and corresponding diagnosis. (PDF)

S2 Table MicroRNA expression in aqueous–full set of data. (XLSX)

S3 Table MicroRNA expression in vitreous–full set of data. (XLSX)

S4 Table MicroRNA expression in plasma–full set of data. (XLSX)

S5 Table Unique microRNAs in aqueous samples. (DOCX)

S6 Table Unique microRNAs in vitreous samples. (DOCX)

S7 Table Unique microRNAs in plasma. (DOCX)
