# Supplementary material for: Unique molecular signatures of microRNAs in ocular fluids and plasma in diabetic retinopathy
Source: PLoS One. 2020 Jul 21;15(7):e0235541. doi: 10.1371/journal.pone.0235541 (PMC7373301; doi:10.1371/journal.pone.0235541)
Supplement: S1 Table — (PDF) [file pone.0235541.s001.pdf]

Supplementary Table 1

	Sample			Type	Group	Diagnosis	POH	Dialysis	Creatine	PRP	Injections	Lens	Intact capsule (if PCIOL)
	**CONTROLS**												
1	AQC-11	VIT-11	PLS-11	RD	CON	RD repair OD	retinal detachment						
2	AQC-19	VIT-19	PLS-19	MH-OS	CON	MH OS							
3	AQC-32	VIT-32	PLS-32	ERM	CON	ERM OU							
4	AQC-33	VIT-33	PLS-33	ERM	CON	ERM OD							
5	AQC-39	VIT-39	PLS-39	ERM	CON	ERM OS							
6	AQC-41	n/a	PLS-41	ERM	CON	PVR, explant of retisert implant,							
7	AQC-43	VIT-43	PLS-43	ERM	CON	ERM OS							
8	AQC-44	VIT-44	PLS-44	ERM	CON	ERMS OS							
9	AQC-47	VIT-47	PLS-47	ERM	CON	ERM OD							
10	AQC-52	VIT-52	PLS-52	ERM	CON	ERM OD							
11	n/a	VIT-53	n/a		CON		Optic nerve pit, congenital						
	**DIABETIC RETINOPATHY - TYPE I (DMI-PDR)**												
1	AQC-21	VIT-21	PLS-21	IDDM (I)	DR-Type I	VH OS	PDR OU	N	1.14	PRP		Phakic	
2	AQC-26	VIT-26	PLS-26	IDDM (I)	DR-Type I	VH OS	PDR OU	N	2.45	PRP		Phakic	
3	AQC-27	VIT-27	PLS-27	IDDM (I)	DR-Type I	TRD OS	PDR OU	N	0.69	PRP	Avastin	Phakic	
4	AQC-31	VIT-31	PLS-31	IDDM (I)	DR-Type I	TRD OS	PDR OU	N	2.60	PRP		Phakic	
5	n/a	n/a	PLS-63	IDDM (I)	DR-Type I		PDR, no DME						
	**DIABETC RETINOPATHY - TYPE II - PROLIFERATIVE (DMII-PDR)**												
1	AQC-17	VIT-17	PLS-17	IDDM (II)	DR-Type II	TRD repair OD	PDR OU	N	1.61	PRP		Phakic	
2	AQC-24	VIT-24	PLS-24	IDDM (II)	DR-Type II	VH OS	PDR OU	N	0.57			Phakic	
3	AQC-40	VIT-40	PLS-40	IDDM (II)	DR-Type II	VH OS	PDR OU	N	?	PRP		Phakic	
4	AQC-42	VIT-42	PLS-42	IDDM (II)	DR-Type II	VH OS	PDR OU	N	2.27	PRP		Phakic	
5	AQC-46	VIT-46	PLS-46	IDDM (II)	DR-Type II	TRD OS	PDR OU, TRD OU	Pre dialysis	4.09			Phakic	
6	AQC-49	n/a	PLS-49	IDDM (II)	DR-Type II	TRD OS	PDR OU, TRD OS	N	1.51	PRP		Phakic	
7	n/a	VIT-18	n/a	IDDM (II)	DR-Type II	TRD repair OS	PDR OU	Pre dialysis	4.07			Phakic	
	**DIABETIC RETINOPATHY - TYPE II (DMII-NPDR)**												
1	AQC-12	VIT-12	PLS-12	NIDDM (II)	DR-Type II	ERM OD	NPDR OU	N	1.02		Avastin	Phakic	
2	AQC-28	VIT-28	PLS-28	IDDM (II)	DR-Type II	VMT OS	NPDR no CSME OU	N	0.97			PCIOL	yes
3	AQC-58	VIT-58	PLS-58	IDDM (II)	DR-Type II								
4	AQC-71	VIT-71	PLS-71		DR-Type II		NPDR,DME						
