# Supplementary material for: Unique molecular signatures of microRNAs in ocular fluids and plasma in diabetic retinopathy
Source: PLoS One. 2020 Jul 21;15(7):e0235541. doi: 10.1371/journal.pone.0235541 (PMC7373301; doi:10.1371/journal.pone.0235541)
Supplement: S5 Table — (DOCX) [file pone.0235541.s005.docx]

**S5 Table DMI-PDR UNIQUES**

| **AQC-DMI-PDR (25)** | **FC** | **VIT-DMI-PDR (50)** | **FC** | **PLS-DMI-PDR (20)** | **FC** |
| --- | --- | --- | --- | --- | --- |
| hsa-miR-3202_st  hsa-miR-1267_st  hsa-miR-29a_st  hsa-miR-4665-5p_st  hsa-miR-26b-star_st  hsa-miR-548c-3p_st  hsa-miR-4677-5p_st  hsa-miR-4699-3p_st  hsa-miR-3121-5p_st  hsa-miR-3611_st  hsa-miR-137_st  hsa-miR-1973_st  hsa-miR-3657_st  hsa-miR-4536_st  hsa-miR-3064-3p_st  hsa-miR-216a_st  hsa-miR-3157-5p_st  hsa-miR-384_st  hsa-miR-624_st  hsa-miR-4314_st  hsa-miR-4506_st  hsa-miR-31_st  hsa-miR-3659_st  hsa-miR-518c_st  hsa-miR-548c-5p_st | 1.38  1.35  1.31  1.3  1.27  1.27  1.23  1.23  1.22  1.22  1.21  1.21  1.2  1.2  -1.2  -1.21  -1.21  -1.21  -1.22  -1.23  -1.24  -1.25  -1.25  -1.25  -1.26 | hsa-miR-320b_st  hsa-miR-92a_st  hsa-miR-320a_st  hsa-miR-320c_st  hsa-miR-185_st  hsa-miR-4488_st  hsa-miR-221_st  hsa-miR-423-5p_st  hsa-miR-4695-5p_st  hsa-let-7d_st  hsa-miR-1268b_st  hsa-miR-4499_st  hsa-miR-4484_st  hsa-miR-449c_st  hsa-miR-33a_st  hsa-miR-4429_st  hsa-miR-449b-star_st  hsa-miR-2277-3p_st  hsa-miR-1471_st  hsa-miR-4421_st  hsa-miR-3179_st  hsa-miR-411-star_st  hsa-miR-4431_st  hsa-miR-514_st  hsa-miR-381_st  hsa-miR-1224-5p_st  hsa-miR-3688-3p_st  hsa-miR-4683_st  hsa-miR-3158-5p_st  hsa-miR-4795-3p_st  hsa-miR-551a_st  hsa-miR-3197_st  hsa-miR-34c-5p_st  hsa-miR-512-3p_st  hsa-miR-4732-5p_st  hsa-miR-4803_st  hsa-miR-195-star_st  hsa-miR-30a_st  hsa-miR-628-3p_st  hsa-miR-654-3p_st  hsa-miR-1234_st  hsa-miR-555_st  hsa-miR-298_st  hsa-miR-3126-5p_st  hsa-miR-4517_st  hsa-miR-4469_st  hsa-miR-3910_st  hsa-miR-3622b-5p_st  hsa-miR-3128_st  hsa-miR-3201_st | 9.32  7.71  6.49  6.12  5.91  2.61  1.93  1.8  1.76  1.67  1.55  1.46  1.43  1.34  1.33  1.33  1.31  1.31  1.3  1.3  1.28  1.28  1.28  1.28  1.27  1.26  1.25  1.25  1.24  1.24  1.24  1.23  1.23  1.23  1.22  1.22  1.21  1.21  1.21  -1.22  -1.23  -1.27  -1.28  -1.28  -1.31  -1.35  -1.36  -1.37  -1.54  -4.77 | hsa-miR-574-3p_st  hsa-miR-425-star_st  hsa-miR-2115_st  hsa-miR-28-3p_st  hsa-miR-1911_st  hsa-miR-134_st  hsa-miR-586_st  hsa-miR-107_st  hsa-miR-106b_st  hsa-miR-20a_st  hsa-miR-25_st  hsa-let-7i_st  hsa-miR-451_st  hsa-miR-532-5p_st  hsa-miR-30e_st  hsa-miR-15b_st  hsa-miR-106b-star_st  hsa-miR-20b_st  hsa-miR-222_st  hsa-miR-15a_st | 2.23  1.83  1.51  1.24  1.23  1.21  1.21  -1.55  -2.41  -2.81  -2.82  -4.53  -5.31  -5.5  -6.02  -6.18  -10.32  -12.82  -19.19  -27.56 |
