# Supplementary material for: Unique molecular signatures of microRNAs in ocular fluids and plasma in diabetic retinopathy
Source: PLoS One. 2020 Jul 21;15(7):e0235541. doi: 10.1371/journal.pone.0235541 (PMC7373301; doi:10.1371/journal.pone.0235541)
Supplement: S6 Table — (DOCX) [file pone.0235541.s006.docx]

**S6 Table DMII-PDR UNIQUES**

| **AQC-DMII-PDR (15)** | **FC** | **VIT-DMII-PDR (21)** | **FC** | **PLS-DMII-PDR (7)** | **FC** |
| --- | --- | --- | --- | --- | --- |
| hsa-let-7b_st  hsa-miR-296-3p_st  hsa-miR-563_st  hsa-miR-1185_st  hsa-miR-26a_st  hsa-miR-2681_st  hsa-miR-933_st  hsa-miR-4711-5p_st  hsa-miR-4445-star_st  hsa-miR-593_st  hsa-miR-874_st  hsa-miR-4314_st  hsa-miR-3605-3p_st  hsa-miR-941_st  hsa-miR-518c_st | 3.51  1.28  1.25  1.24  1.23  1.21  1.21  -1.21  -1.22  -1.22  -1.22  -1.24  -1.25  -1.26  -1.29 | hsa-miR-320c_st  hsa-miR-4488_st  hsa-miR-762_st  hsa-miR-3940-5p_st  hsa-miR-4703-3p_st  hsa-miR-642b_st  hsa-miR-1305_st  hsa-miR-512-3p_st  hsa-miR-4668-3p_st  hsa-miR-139-3p_st  hsa-miR-939_st  hsa-miR-3156-3p_st  hsa-miR-3689b-star_st  hsa-miR-384_st  hsa-miR-4715-5p_st  hsa-miR-3163_st  hsa-miR-3674_st  hsa-miR-4284_st  hsa-miR-4712-3p_st  hsa-miR-1273g_st  hsa-miR-3201_st | 3.86  1.79  1.59  1.38  1.29  1.29  1.28  1.27  1.25  1.23  1.23  1.22  1.22  -1.22  -1.22  -1.24  -1.24  -1.25  -1.26  -1.3  -3.83 | hsa-miR-639_st  hsa-miR-1238_st  hsa-miR-3668_st  hsa-miR-569_st  hsa-miR-106a_st  hsa-miR-20a_st  hsa-miR-20b_st | 1.23  1.21  1.21  -1.27  -2.03  -2.05  -8.06 |
