# Supplementary material for: Unique molecular signatures of microRNAs in ocular fluids and plasma in diabetic retinopathy
Source: PLoS One. 2020 Jul 21;15(7):e0235541. doi: 10.1371/journal.pone.0235541 (PMC7373301; doi:10.1371/journal.pone.0235541)
Supplement: S7 Table — (DOCX) [file pone.0235541.s007.docx]

**S7 Table - DMII-NPDR UNIQUE miRNAs for each FLUID**

| **AQC-DRII-NPDR (31)** | **FC** | **VIT-DRII-NPDR (46)** | **FC** | **PLS-DRII-NPDR (56)** | **FC** |
| --- | --- | --- | --- | --- | --- |
| hsa-miR-3201_st  hsa-miR-4667-5p_st  hsa-miR-204_st  hsa-miR-26a_st  hsa-miR-1278_st  hsa-miR-4717-3p_st  hsa-miR-4471_st  hsa-miR-4689_st  hsa-miR-122-star_st  hsa-miR-2115_st  hsa-miR-4687-3p_st  hsa-miR-4697-3p_st  hsa-miR-3116_st  hsa-miR-3685_st  hsa-miR-4423-3p_st  hsa-miR-103a-2-star_st  hsa-miR-4718_st  hsa-miR-3183_st  hsa-miR-4289_st  hsa-miR-4474-5p_st  hsa-miR-377_st  hsa-miR-3159_st  hsa-miR-548al_st  hsa-miR-621_st  hsa-miR-548y_st  hsa-miR-4744_st  hsa-miR-1587_st  hsa-miR-409-5p_st  hsa-miR-4762-5p_st | 1.74  1.42  1.36  1.35  1.32  1.31  1.28  1.27  1.26  1.25  1.24  1.24  1.23  1.22  1.22  1.21  1.21  1.2  1.2  1.2  -1.2  -1.21  -1.22  -1.22  -1.23  -1.25  -1.26  -1.26  -1.26 | hsa-miR-2861_st  hsa-miR-4516_st  hsa-miR-4467_st  hsa-miR-4270_st  hsa-miR-149-star_st  hsa-miR-4281_st  hsa-miR-574-3p_st  hsa-miR-762_st  hsa-miR-3185_st  hsa-miR-4459_st  hsa-miR-3621_st  hsa-miR-3978_st  hsa-miR-1275_st  hsa-miR-466_st  hsa-miR-1272_st  hsa-miR-3688-3p_st  hsa-miR-4490_st  hsa-miR-1273e_st  hsa-miR-572_st  hsa-miR-4758-5p_st  hsa-miR-139-3p_st  hsa-miR-548u_st  hsa-miR-29c_st  hsa-miR-199b-5p_st  hsa-miR-214-star_st  hsa-miR-4770_st  hsa-miR-4685-5p_st  hsa-let-7a_st  hsa-miR-524-3p_st  hsa-miR-4503_st  hsa-miR-1231_st  hsa-miR-3162-5p_st  hsa-miR-3913-3p_st  hsa-miR-3662_st  hsa-miR-4788_st  hsa-miR-3689b-star_st  hsa-miR-4730_st  hsa-miR-3165_st  hsa-miR-4728-3p_st  hsa-miR-4709-3p_st  hsa-miR-766_st  hsa-miR-4707-3p_st  hsa-miR-4509_st  hsa-miR-1281_st | 73.04  3.9  2.79  2.5  1.91  1.83  1.63  1.59  1.57  1.57  1.55  1.43  1.37  1.37  1.36  1.35  1.35  1.34  1.33  1.32  1.31  1.3  1.28  1.27  1.27  1.27  1.26  1.25  1.25  1.24  1.23  1.23  1.23  1.22  1.22  1.21  -1.24  -1.27  -1.27  -1.28  -1.29  -1.3  -1.33  -2.96 | hsa-miR-320b_st  hsa-miR-320c_st  hsa-miR-320a_st  hsa-miR-3960_st  hsa-miR-4787-5p_st  hsa-miR-4754_st  hsa-miR-4672_st  hsa-miR-671-5p_st  hsa-miR-4753-5p_st  hsa-miR-3665_st  hsa-miR-4297_st  hsa-miR-4642_st  hsa-miR-1273d_st  hsa-miR-3064-3p_st  hsa-miR-3646_st  hsa-miR-4302_st  hsa-miR-4431_st  hsa-miR-885-3p_st  hsa-miR-1283_st  hsa-miR-3653_st  hsa-miR-922_st  hsa-miR-100-star_st  hsa-miR-3676_st  hsa-miR-454_st  hsa-miR-183_st  hsa-miR-486-5p_st  hsa-miR-17-star_st  hsa-miR-92b_st  hsa-miR-182_st  hsa-miR-106b_st  hsa-miR-106a_st  hsa-miR-17_st  hsa-miR-16_st  hsa-miR-18b_st  hsa-miR-500a-star_st  hsa-miR-15b_st  hsa-miR-502-3p_st  hsa-miR-25_st  hsa-miR-486-3p_st  hsa-let-7i_st  hsa-miR-20a_st  hsa-miR-660_st  hsa-miR-192_st  hsa-miR-27a_st  hsa-miR-150_st  hsa-miR-30e_st  hsa-miR-194_st  hsa-miR-363_st  hsa-let-7g_st  hsa-miR-451_st  hsa-miR-106b-star_st  hsa-miR-18a_st  hsa-miR-15a_st  hsa-miR-20b_st | 2.37  2.09  2.05  1.58  1.4  1.29  1.25  1.25  1.24  1.23  1.23  1.23  1.22  1.21  1.21  1.21  1.21  1.21  1.2  1.2  -1.2  -1.21  -1.22  -1.23  -1.45  -1.61  -1.69  -1.71  -1.83  -2.03  -2.07  -2.23  -2.33  -2.38  -2.43  -2.61  -2.68  -2.87  -2.98  -3.22  -3.81  -4.06  -4.28  -4.52  -4.66  -5.72  -8.32  -8.42  -9.32  -9.88  -10.6  -11.31  -25.27  -39.65 |
